# Design of a Novel Medical Acoustic Sensor Based on MEMS Bionic Fish Ear Structure

**DOI:** 10.3390/mi13020163

**Published:** 2022-01-22

**Authors:** Chenzheng Zhou, Junbin Zang, Chenyang Xue, Yuexuan Ma, Xiaoqiang Hua, Rui Gao, Zengxing Zhang, Bo Li, Zhidong Zhang

**Affiliations:** State Key Laboratory of Dynamic Measurement Technology, North University of China, Taiyuan 030051, China; s1906213@st.nuc.edu.cn (C.Z.); myx59986686@163.com (Y.M.); huaxq1128@163.com (X.H.); 18406583750@163.com (R.G.); zhangzengxing@nuc.edu.cn (Z.Z.); lb@nuc.edu.cn (B.L.); zdzhang@nuc.edu.cn (Z.Z.)

**Keywords:** bionic, electronic stethoscope, heart sound, low-frequency, MEMS applications, MEMS sensors, micromachine, simulations

## Abstract

High-performance medical acoustic sensors are essential in medical equipment and diagnosis. Commercially available medical acoustic sensors are capacitive and piezoelectric types. When they are used to detect heart sound signals, there is attenuation and distortion due to the sound transmission between different media. This paper proposes a new bionic acoustic sensor based on the fish ear structure. Through theoretical analysis and finite element simulation, the optimal parameters of the sensitive structure are determined. The sensor is fabricated using microelectromechanical systems (MEMS) technology, and is encapsulated in castor oil, which has an acoustic impedance close to the human body. An electroacoustic test platform is built to test the performance of the sensor. The results showed that the MEMS bionic sensor operated with a bandwidth of 20–2k Hz. Its linearity and frequency responses were better than the electret microphone. In addition, the sensor was tested for heart sound collection application to verify its effectiveness. The proposed sensor can be effectively used in clinical auscultation and has a high SNR.

## 1. Introduction

Sound is closely related to our lives. All living things in nature vibrate and make sounds. Humans have long used acoustic principles to identify physiological and pathological sounds which are produced in the body [1]. Therefore, it is of great significance to medical diagnosis that the sounds produced by various organs of the human body can be accurately acquired and analysed. With the development of medical technology, the demand for non-invasive and high-precision detection equipment has greatly increased, which relies on high-performance sensors. Acoustic diagnostic instruments have become a hot area of current research due to their non-invasiveness and diagnostic mechanism [2]. High-performance medical acoustic sensors are the core of the instrument and the key to this research.

The acoustic signals of the human body include the heart, lungs, bowels and other sound signals [3]. These signals are extremely weak but contain a lot of health-related physiological and pathological information. In order to better capture these weak acoustic signals, Rene Laennec invented a mechanical stethoscope, which can non-linearly amplify the collected sounds [4]. With the development of technology, it has been found that mechanical stethoscopes are not ideal, due to the resonance effect of the catheter. Their frequency responses have extreme values at some frequencies, and different models’ transmission characteristics are quite different [5]. Therefore, 3M Corporation invented the electronic stethoscope. The electronic stethoscope uses electronic technology to amplify the sounds and overcome the high noise of mechanical stethoscopes. It converts sound waves into electrical signals through sensors and then amplifies and processes them to obtain the best results. As the core of the stethoscope, the sensor determines the stethoscope’s performance [6]. At present, the most widely used acoustic sensors are mainly divided into two types: electret capacitive sensors and piezoelectric sensors [7]. The acoustic signals have to pass through the air medium during the transmission between the body and the transducer, resulting in a high loss of acoustic energy. Electret capacitive sensors are often used in microphones and have the advantage of low cost, but the SNR is poor [8]. Piezoelectric sensors are mainly used in ultrasonic testing [9]. Although they have good electromechanical coupling coefficients, the similarity between the heart sound signals extracted by the piezoelectric electronic stethoscope and the original signals is less than 70%, increasing the difficulty for doctors’ judgments [10].

As a new science involving multiple disciplines, bionics has been providing a steady stream of vitality for engineering science innovation, solving many critical scientific and technological problems in engineering applications, and accelerating the development of modern science and technology. At present, many powerful acoustic instruments in different fields adopt bionic designs, such as radar and sonar. MEMS technology can be applied to designing and manufacturing complex bionic sensing structures [11]. Xue et al. proposed a novel MEMS bionic vector hydrophone based on the principle that fish’s lateral line organs sense water flow pressure and flow velocity. It can be well applied to underwater detection and has a good frequency response in low frequency and sensitivity [12,13]. Afterwards, Zhang et al. optimised and improved this model [14,15], and Liu et al. proposed a lollipop-shaped hydrophone that improves the sensitivity [16]. Li et al. applied cilium-shaped and lollipop-shaped hydrophones to heart sound detection and compared their performance [17]. Duan and Cui et al. further improved the cilium structure, designed a bat-shaped structure, and conducted experimental verification [18,19].

Similarly, the fish’s ear has undergone a long evolutionary process and has an unparalleled advantage in obtaining sound signals. Sound travels faster in water than air. Fish are full of liquid, and thus the sound can pass directly through their bodies to their ears. Due to the special structure of fish ears, they have the advantage of perceiving low-frequency sounds and hearing sounds that humans cannot hear. Therefore, it is essential to study the sound propagation mechanism of fish ears and develop acoustic sensors with bionic structures.

With the research on bionics and fish ear structure, the sound transmission mechanism of fish ears has a more scientific theoretical support at the micro-level [20,21]. MEMS technology provides technical feasibility for manufacturing medical bionic microstructures and developing high-performance medical sensors. The acoustic diagnosis technology has made tremendous developments. Among them, ultrasonic diagnostic equipment has made significant progress in three-dimensional imaging, digital data acquisition, processing technology, etc. However, improving the resolution of the ultrasound examination is still a challenge faced by researchers [22]. As the most widely used diagnosis method, auscultation has unrivalled advantages, such as convenience, low cost, and high efficiency. The stethoscope can solve the main content required for physical diagnosis in the clinic. Modern medical diagnosis methods such as X-ray, electrocardiogram, echocardiogram, and radiography cannot completely replace it [23]. This paper designs a medical acoustic sensor with the fish ear structure based on MEMS technology by studying the sensing and bionic mechanism, which needs experimental verification. The results show that the sensor has good acoustic performance and can be applied to detect human heart sound signals.

## 2. Materials and Methods

### 2.1. Sensor Principle and Optimisation Design

**Figure 1:**
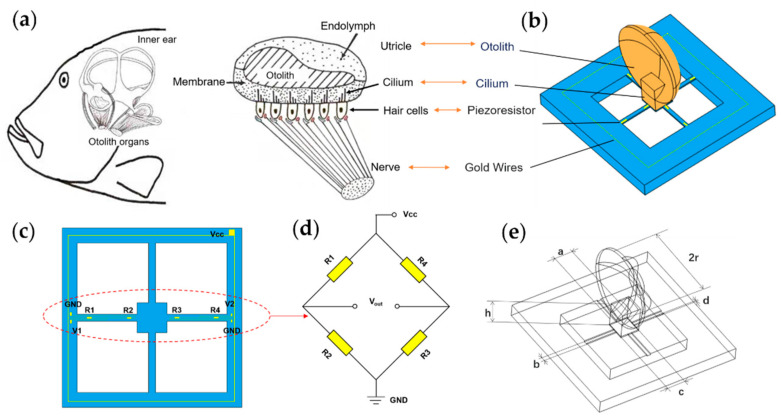
The principle of microstructure. (**a**) The schematic view of the fish ear structure. (**b**) The microstructural model of the bionic MEMS sensor. (**c**) Distribution and connection of the piezoresistors. (**d**) The Wheatstone circuit diagram. (**e**) The illustration of microstructure parameters.

The structure of the fish ear is shown in Figure 1a. Unlike the human ear structure, fish only have the inner ear. The inner ear helps fish perceive the pressure waves caused by sound, and the displacement of the water in its flow, both to enable them to obtain auditory signals and help them maintain their balance. The main structure of the fish ear is the utricle. Fish receive sound waves through the utricle, which causes the cilium of hair cells to oscillate, generating nerve signals transmitted to the brain, forming hearing [24,25]. Based on this process, a bionic MEMS acoustic sensor is designed as the core component of the MEMS electronic stethoscope, as shown in Figure 1b.

**Figure 2:**
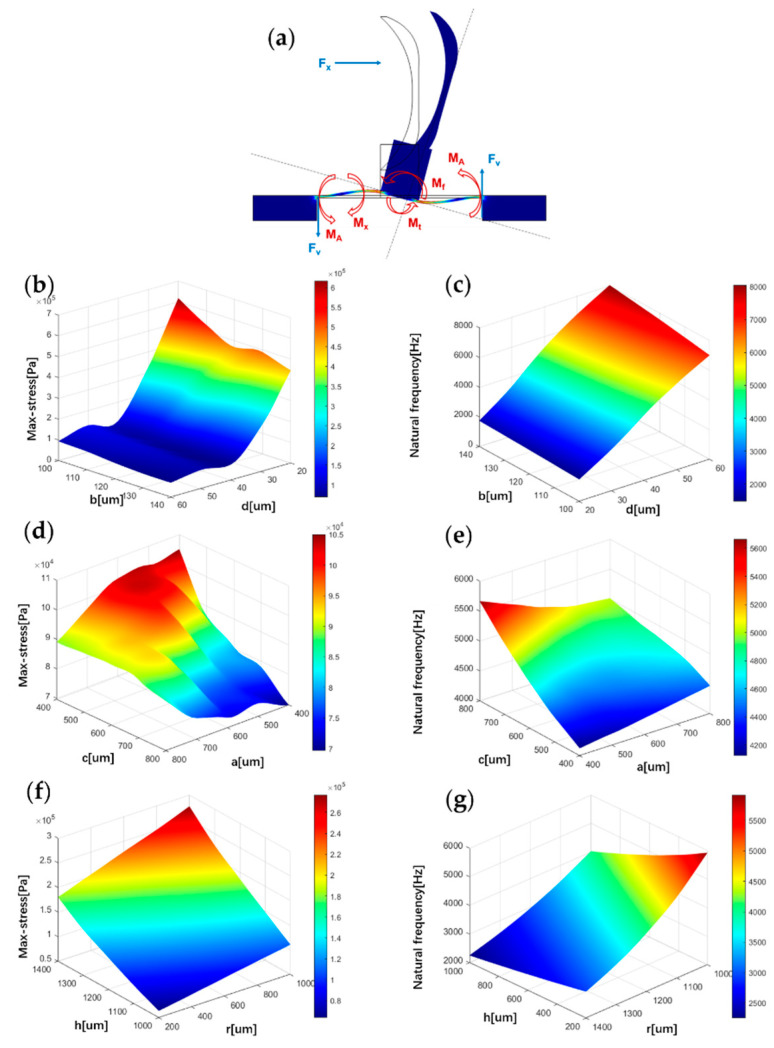
Influence of structure parameters on stress and natural frequency. (**a**) Microstructure force analysis. (**b**) The correlation between maximum stress on the beam and parameters *b*, *d*. (**c**) The correlation between natural frequency and parameters *b*, *d*. (**d**) The correlation between maximum stress on the beam and parameters *c*, *a*. (**e**) The correlation between natural frequency and parameters *c*, *a*. (**f**) The correlation between maximum stress on the beam and parameters *h*, *r*. (**g**) The correlation between natural frequency and parameters *h*, *r*.

The bionic acoustic sensor consists of cantilever beams and a fish ear micromechanical structure design, which is manufactured by MEMS technology, as shown in Figure 1c. The cantilever beam is covered with piezoresistors. Four piezoresistors form a Wheatstone bridge, as shown in Figure 1d. When the otolith structure senses sound waves and vibrations under force, these vibrations are transmitted through the cilium structure to the central mass block to which the cantilever beam is attached, causing the deformation of the beams, as shown in Figure 2a. The Wheatstone bridge converts this deformation into a differential voltage signal output [26,27]. The above process simulates the process of the fish ear from receiving sound waves to outputting neural signals.

When acoustic waves act on the bionic structure, under the action of flexural moment $M_{x}$ produced by the *x*-axis direction and the horizontal force $F_{x}$, the stress at any point x on the cantilever beams can be calculated from the following expression [16]:

(1) $$\sigma_{\left(x\right)}=\pm \frac{l^{2}+1.5c\left(l-x\right)+3xl}{\frac{2}{3}bd^{2}(l^{2}+1.5cl+\frac{3}{4}c^{2})}M_{x}\pm \frac{F_{x}}{bd}$$

the parameters b, c, d and l are defined in Figure 1e and Table 1.

Under the action of stress, the value of the piezoresistors on the cantilever beams will change, and the change ΔR can be calculated from the following expression [19]:

(2) $$\frac{\Delta R}{R}=(1+2\mu )\varepsilon +\pi_{1}E\varepsilon \approx \pi_{1}E\varepsilon =\pi_{1}\sigma$$

where $\pi_{1}$ is the piezoresistive coefficient of the Si material, E is the elastic modulus and σ is the stress on the material.

At the same time, the Wheatstone bridge outputs the voltage generated by the change in piezoresistors:

(3) $$V_{out}=\left(\pm \frac{l^{2}+1.5c\left(l-x\right)+3xl}{\frac{2}{3}bd^{2}\left(l^{2}+1.5cl+\frac{3}{4}c^{2}\right)}\cdot \frac{\left(h+2r\right)}{2}\pm \frac{1}{bd}\right)\pi_{1}V_{in}F_{x}$$

where $V_{in}$ denotes the input voltage.

It can also be expressed as:

(4) $$V_{out}=\frac{\left(R_{1}+\Delta R_{1})(R_{3}+\Delta R_{3})-(R_{2}-\Delta R_{2})(R_{4}-\Delta R_{4}\right)}{\left(R_{1}+\Delta R_{1}+R_{2}-\Delta R_{2})(R_{3}+\Delta R_{3}+R_{4}-\Delta R_{4}\right)}V_{in}$$

where $R_{1}$, $R_{2}$, $R_{3}$ and $R_{4}$ denote the four piezoresistors. $\Delta R_{1}$, $\Delta R_{2}$, $\Delta R_{3}$, $\Delta R_{4}$ denote the resistance variation of $R_{1}$, $R_{2}$, $R_{3}$ and $R_{4}$, respectively.

Heart sound signals are weak signals with frequencies of less than 1000 Hz. In order to detect these signals, the sensor must improve the sensitivity and the natural frequency. The finite element simulation of the structure is carried out to optimise the sensor’s performance. The sensor’s sensitivity is related to the stress it is subjected to, and the bandwidth is associated with the first-order natural frequency. In this paper, the maximum stress and the first-order natural frequency represent the effects of different structural parameters on the sensitivity and bandwidth of the sensor, respectively. The acoustic loads acting on the sensor are simplified in the simulation process: the sensitive structure is subjected to an acoustic pressure ∆p in one direction. In the static-state analysis of the sensor, an acoustic pressure of 1 Pa is applied to the fish ear structure. Figure 2b,d,f shows the correlation between maximum stress on the beam and microstructure parameters b, d, c, a, h and r. It was found that as the parameters b and d decrease, the maximum stress on the beam increases. These results fit well with Equation (1). At the same time, this paper also found that the maximum stress on the beam increased as the parameters h and r increase. When c and a are equal, local extremums of maximum stress exist.

In addition to analysing the sensitivity of the bionic microstructure, structural parameters need to be optimised to design a bionic sensing structure that meets its bandwidth acquisition requirements for the specific bandwidth limitation of the heart sound signals [28]. The sensor’s bandwidth is closely related to the natural frequency of the microstructure, which can be expressed as shown in Equation (5).

(5) $$f=\frac{1}{2\pi }\sqrt{\frac{K}{m}}=\frac{1}{2\pi }\sqrt{2\frac{Ebd^{3}}{mlh^{2}}\left(\frac{c^{2}}{l^{2}}+\frac{c}{l}+\frac{1}{3}\right)}$$

where K is the stiffness of the beam, E is the elasticity modulus of the material, and m is the mass of the bionic structure.

The relationship between the natural frequency and various microstructural parameters is visualised in Figure 2c,e,g. It can be found that as the parameters d, and c growth, the natural frequency increases. As the parameters a, h and r growth, the natural frequency decreases.

Based on simulation results and the feasibility of microfabrication, the optimal microstructure parameters are selected in this paper to ensure that the sensor has a higher sensitivity while maintaining a suitable bandwidth. The parameters chosen for this paper are shown in Table 1.

**Figure 3:**
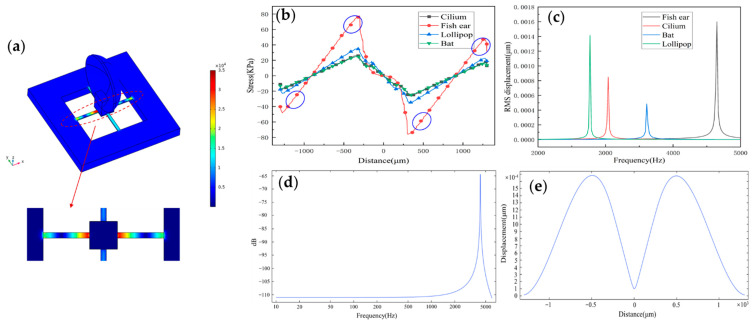
(**a**) The stress distribution on the microstructure. (**b**) The stress distribution curve on the X-direction beam of the sensors under different structures and the placement area of piezoresistors. (**c**) The natural frequency of sensors under different structures. (**d**) The frequency response of the structure (the natural frequency of the sensor is 4239.8 Hz). (**e**) The displacement of the beam.

Figure 3a shows the microstructure stress distribution maps depending on the selected parameters. Figure 3b shows the stress distributions on the beams of four published structures. Values of the *x*-axis indicate the corresponding position on the beam. Compared to the case of the previous structures such as Cilium, Lollipop, and Bat types, the maximum stress of the fish ear is obviously higher, which means that the sensor has high sensitivity. The regions with the highest stresses are distributed at the ends of the beam. Piezoresistors are placed in these regions to improve the sensitivity of the sensor.

Figure 3c shows the natural frequency values of four published structures. All four sensors’ operating frequency bandwidths cover the frequency range of the heart sound signals, which the fish ear sensor has a higher natural frequency. Moreover, the natural frequency of the sensor is 4239.8 Hz. Figure 3d shows the structure’s frequency response by simulation. The sensor has a good flatness in the frequency band 10–1000 Hz, which is consistent with the experimental results. The peak points correspond to the sensor’s natural frequency value. Figure 3e shows the displacement of the beam, and it has good symmetry.

### 2.2. Fabrication Process and Encapsulation

**Figure 4:**
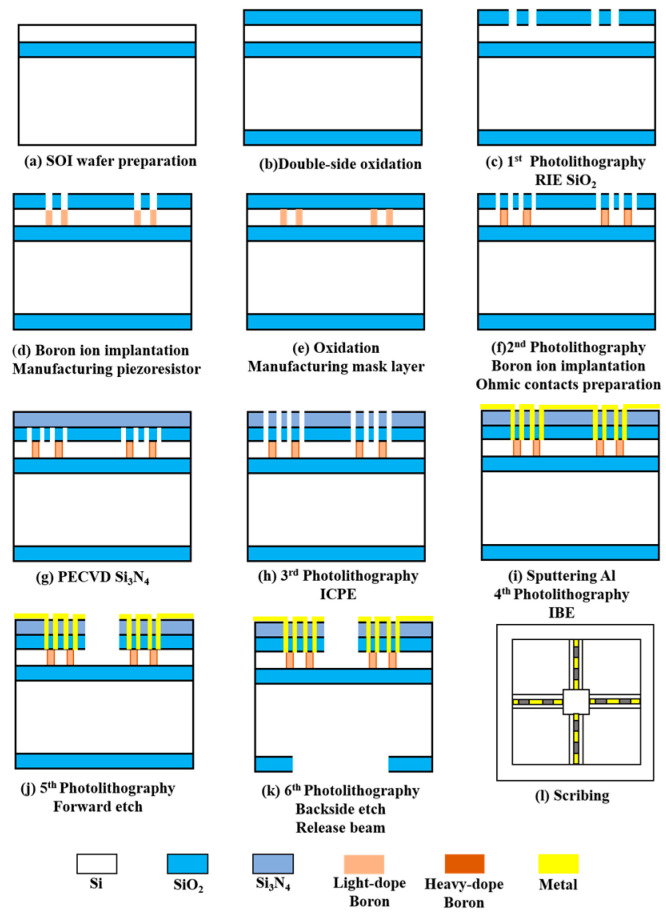
The fabrication process of the sensor.

Based on the finite element simulation results, the fabrication process of the sensor is carried out. The microstructure is fabricated using the Silicon-On-Insulator (SOI). The single-crystal silicon structure has excellent mechanical properties. It can be used to manufacture the connection structure of the cantilever beam and centre mass block. At the same time, the oxide layer as the sacrificial and insulating layer has excellent corrosion-stopping capability, and it is easy to obtain complete, defect-free, uniform thickness and precisely controlled structural layers in the MEMS processing. The devices processed with SOI can effectively reduce parasitic capacitance and power consumption [7]. The fabrication process flow is shown in Figure 4.

**Figure 5:**
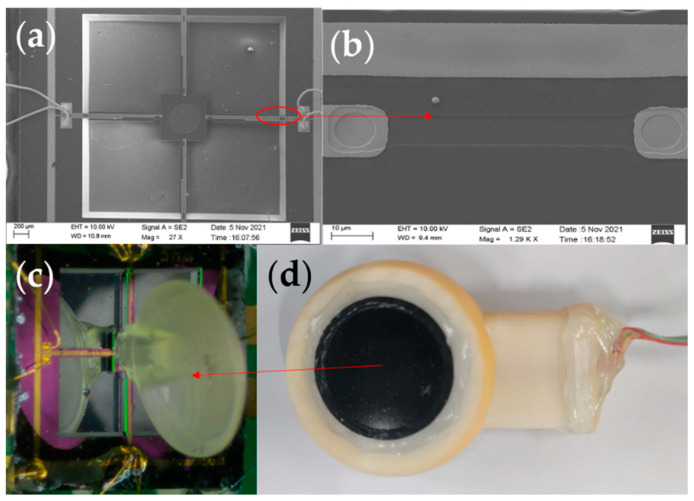
(**a**) SEM image of the overall sensor structure. (**b**) Local SEM image of the piezoresistor. (**c**) Integrated diagram of fish ear structures sensor. (**d**) The sensor probe encapsulation.

First, the SOI wafer was prepared (step a). Then, cleaned and oxidised (step b). The oxide layer was lithographed with RIE (step c), followed by a boron ion injection to form a piezo-resistor region (step d). The second oxidation was performed to form a mask layer (step e). The second lithography was performed to form an ohmic contact region, and a high concentration of boron ion was injected again (step f). PECVD was used to precipitate Si_3_N_4_ (step g), and ICPE was used to etch oxidation holes (step h). Sputtering metallic aluminium was used to form metal leads (step i). Finally, RIE was used to etch the cantilever beam structure (step j). The backside etching was performed using deep silicon etching to release the beam structure (step k). Figure 5a shows an SEM image of the overall sensor structure. Figure 5b shows the local SEM image of the piezoresistor. The fish ear microstructure was fabricated using Projection Micro Stereolithography (PμSL) technology. Its integrations with the cantilever beams were integrated by UV curing. The 3D control platform allowed precise control of the integration process, ensuring precise bonding and consistency between the sensors. The sensitive structure of the sensor after integration is shown in Figure 5c.

When sound is transmitted in different media, the greater the difference in impedance between the two media, the greater the acoustic reflection coefficient and the energy loss of the sound. The fish are very sensitive to sound as their bodies contain a lot of fluid, and sound can pass right through their bodies and reach their ears. For the above reasons, in order to better simulate the transmission process of sound in the fish ear and at the same time ensure that the attenuation of the signal in different media is as small as possible, castor oil was chosen as the transmission medium between the human body and the sensor [29]. Table 2 shows some material parameters. At the same time, expanded PTFE (E-PTFE) material was chosen as the membrane for the sensor probe. This material has good water resistance and excellent air permeability, with a surface of 0.1–0.25 μm microporosity. It also provides a better fit to the surface skin of the human body. Therefore, it has excellent sound transmission. The sensor probe encapsulation diagram is shown in Figure 5d.

## 3. Results

### 3.1. Acoustic Properties Testing

**Figure 6:**
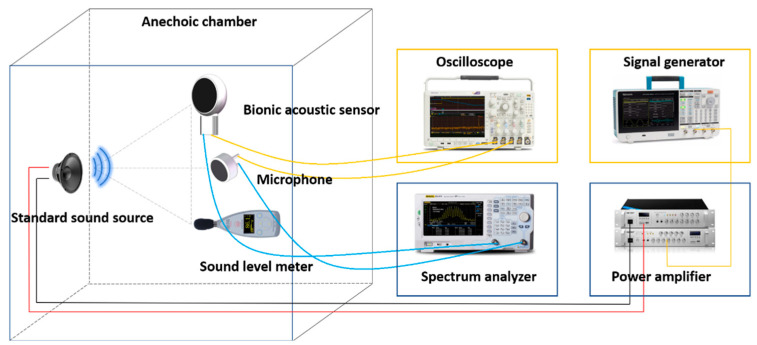
Diagram of the test platform.

After the encapsulation process of the sensor, this paper tested the performance of the sensor. The acoustic test platform was set up to test the acoustic performance. The diagram of the test platform is shown in Figure 6. The anechoic chamber can be well insulated from ambient noise, and a standard sound source (RMS.50 W, MAX.180 W) was placed in it. The MEMS bionic acoustic sensor, sound pressure meter, and sound source were placed in an isosceles triangle, while an electret microphone was set as a reference [30]. The test method was as follows:

1. The signal generator (Tektronix 31052) sent a standard sinusoidal signal, which was amplified by a power amplifier and transmitted to the standard sound source to form acoustic waves;
2. The MEMS bionic sensor and the electret microphone converted the acoustic signal into an electrical signal and output the waveform to the oscilloscope (Tektronix MSO64B). The spectrum analyser analysed the current signal in the frequency domain. The sound level meter displayed the sound pressure level in the current environment;
3. The frequency and amplitude of the sinusoidal signal were changed by adjusting the power amplifier and signal generator to control the sound pressure variation. Then, we recorded the output voltage and frequency response of the MEMS bionic sensor and the electret microphone.

**Figure 7:**
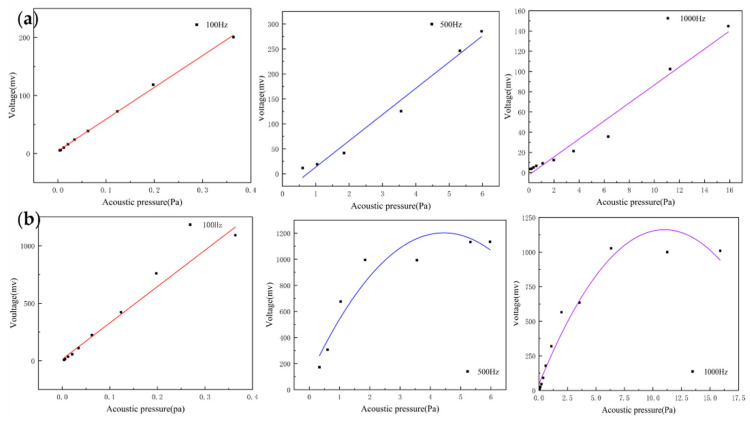
(**a**) The linearity of the MEMS bionic sensor at 100 Hz, 500 Hz, and 1000 Hz. (**b**) The linearity of the electret microphone at 100 Hz, 500 Hz, and 1000 Hz.

For acoustic sensors, linearity is an important indicator and can be assessed by recording the output voltage of the sensors at different sound pressures. They can indicate the input–output characteristic of the sensor. The heart sound signal is weak with very low sound pressure. If the output response of the sensor is non-linear, this will result in the inaccurate amplitude of the acquired heart sound signals. This creates a large deviation in the amplitude and energy of the acquired heart sound signal compared with the actual one, which affects the subsequent data analysis. By testing whether the linear range of the sensor covers the frequency band requirements of the measured signal, the rationality and effectiveness of the structure can be verified. The MEMS bionic acoustic sensor showed an excellent linear response with an R-squared value of 0.99. This is shown in Figure 7a, and the linearity of the electret microphone is shown in Figure 7b. It was found that the linearity of the MEMS bionic sensor was more stable at different frequencies and was better than the electret microphone at 500 Hz and 1000 Hz. This result shows that the MEMS bionic sensor outperformed the electret microphone in the low-frequency band.

**Figure 8:**
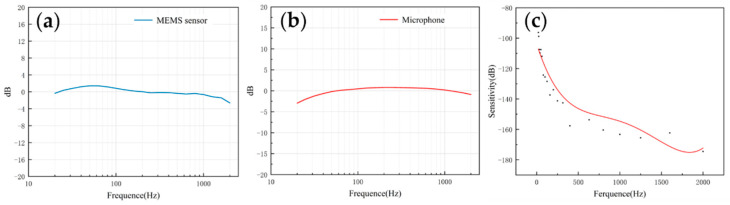
(**a**) The frequency response of the MEMS bionic sensor. (**b**) The frequency response of the electret microphone. (**c**) The sensitivity of the MEMS bionic sensor.

The frequency response describes the sensor’s ability to process signals at different frequencies. Since the heart sound signal is mainly in the range of 20–600 Hz and the breath sound signal is mainly below 1000 Hz, this paper mainly tested the frequency response of the two sensors in the range of 20–2000 Hz. As shown in Figure 8a,b, both of them had a flat frequency response, which was very close to the simulation results. The flatness of the microphone and the MEMS bionic acoustic sensor were 3.41 dB and 1.76 dB. The MEMS bionic sensor was better than the electret microphone at 20–100 Hz.

The sensitivity can be used to evaluate the ability of the sensor to pick up weak signals, and is expressed as Equation (6). The sensitivity was calibrated by the test platform, and the results are shown in Figure 8c. The sensitivity of the sensor achieved −163.3 dB (re:1 V/μPa) at 1000 Hz. In the low-frequency band, the MEMS bionic acoustic sensor had good characteristics.

(6) $$S_{n}=\frac{\Delta y}{\Delta x}$$

where Δx is the variation of the sound pressure, and Δy is the variation of the output voltage.

### 3.2. Heart Sound Detection

**Figure 9:**
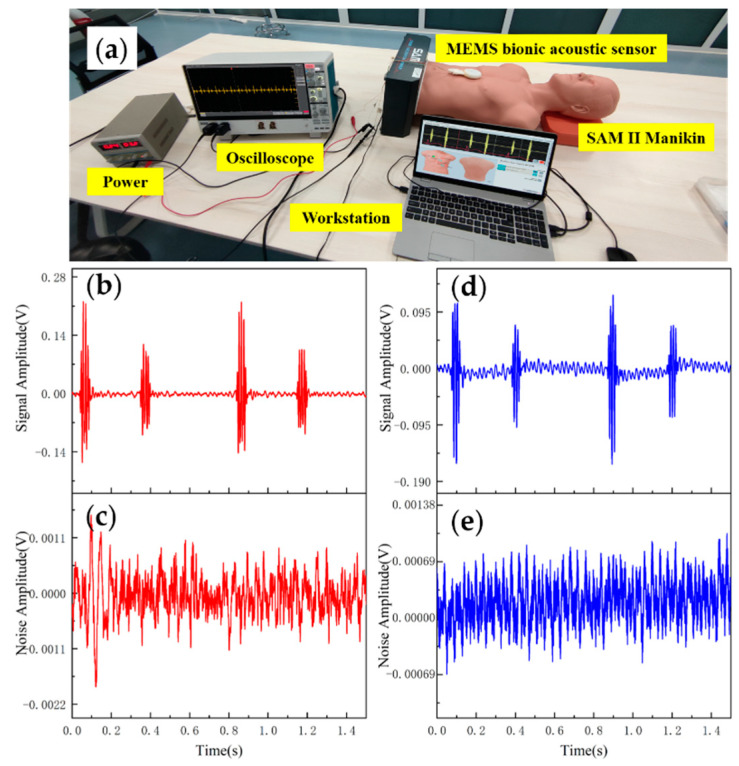
(**a**) Heart sound detection test. (**b**) Normal heart sound signal collected by MEMS sensor. (**c**) The noise of MEMS sensor. (**d**) Normal heart sound signal collected by 3M electronic stethoscope. (**e**) The noise of 3M electronic stethoscope.

Data analysis and evaluation of the collected signals are essential for the sensor’s practicability. After completing the acoustic performance tests, the sensor was tested for application. The test was conducted using 3B Scientific™ SAM II^®^ Student Auscultation Manikin. It contained nearly 100 kinds of high-quality physiological and pathological heart, respiratory and intestinal sounds. Students could auscultate with an acoustic stethoscope without noise interference. The MEMS bionic acoustic sensor and 3M™ Littmann^®^ 3200 electronic stethoscope (3M electronic stethoscope) were used to collect heart sound signals. Firstly, a normal heart sound signal with a heart rate of 75 bpm was collected. The waveform is shown in Figure 9. It can be seen that the normal heart sound signal collected by the MEMS sensor had obvious characteristics. The first heart sound (S1) and the second heart sound (S2) were clearly visible, and the heartbeat period was 0.8 s. According to Equation (7) and Table 3, the SNR of the MEMS sensor and the 3M electronic stethoscope were 38.6 dB and 37.6 dB, respectively, with the MEMS sensor being slightly better than the 3M electronic stethoscope by 1 dB.

(7) $$SNR=20\log(\frac{v_{s}}{v_{n}})$$

where $v_{s}$ is the effective voltage value of the heart sound signal, and $v_{n}$ is the effective voltage value of the noise.

Table 4 shows the comparisons of SNR of sensors with different structures. The fish ear structure had the highest SNR. The above experimental results demonstrated that both sensors could effectively capture the normal heart sound signal. The normal heart sound signal consisted mainly of S1 and S2. They marked the beginning and end of the ventricular contractions, respectively, which can be easily captured by stethoscopes. The third (S3) and fourth (S4) heart sounds differed from them in that they were low-frequency sounds, fainter and more difficult to capture by stethoscopes. The frequencies of S3 were mainly around the frequency bands of 50–70 Hz, and the S4 are mainly around 50–70 Hz and 150–170 Hz [31,32]. They were produced by ventricular filling rather than being associated with valve closure. In this paper, to verify the ability to collect low-frequency signals, the MEMS sensor was used to collect S3 and S4. The acquired waveforms were compared with that of the 3M electronic stethoscope.

**Figure 10:**
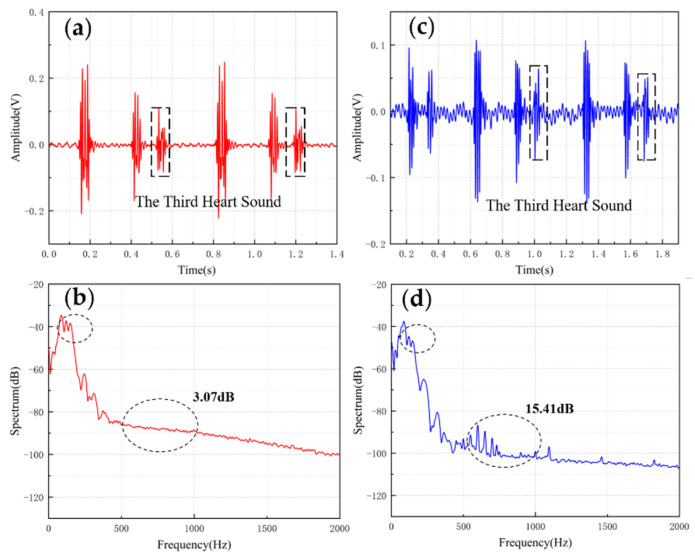
(**a**) The S3 waveform collected by MEMS sensor. (**b**) The spectrum of the signal from MEMS sensor. (**c**) The S3 waveform collected by 3M electronic stethoscope. (**d**) The spectrum of the signal from 3M electronic stethoscope.

**Figure 11:**
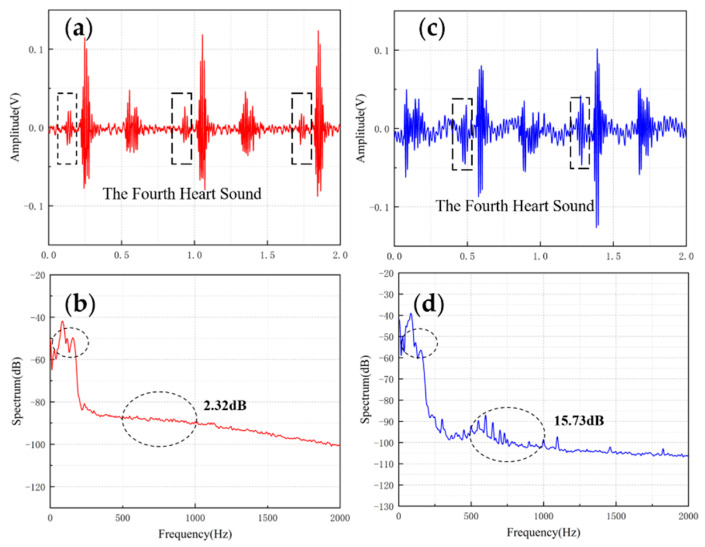
(**a**) The S4 waveform collected by MEMS sensor (**b**) The spectrum of the signal from MEMS sensor (**c**) The S4 waveform collected by 3M electronic stethoscope (**d**) The spectrum of the signal from 3M electronic stethoscope.

The results are shown in Figure 10 and Figure 11. From the time domain, both sensors can capture S3 and S4, but the signals from the MEMS sensor had a higher SNR. From the frequency domain, the spectrums of the S3 (50–70 Hz) and S4 (50–70 Hz) collected by both sensors were close, but the S4 (150–170 Hz) collected by the MEMS sensor was better than 3M electronic stethoscope 7 dB. The main energy of the heart sound signal was concentrated below 600 Hz, and 90% of the energy was concentrated in the range 20–150 Hz. This indicates that the heart sound signal collected by this MEMS sensor can cover its theoretical range completely. At the same time, the 3M electronic stethoscope picked up some components at 500–1000 Hz, while the MEMS sensor’s spectrum was flatter. The fluctuation range of the MEMS sensor was 3.07 dB and 2.32 dB, and the 3M electronic stethoscope was 15.41 dB and 15.73 dB. In summary, the time-frequency characteristics of the heart sound signals collected by the MEMS sensor were essentially the same as the signal collected by the 3M electronic stethoscope, even better than it in some ways. As a result, the proposed sensor can collect accurate and valid heart sound signals, and be used in auscultation.

## 4. Conclusions

This paper innovatively proposes a MEMS acoustic sensor imitating fish ears. The sensor’s microstructure is designed by studying the mechanism of the fish ears’ mechanism to feel sound in water. The microstructure parameters are optimised by finite element simulation, and the sensor’s sensitivity, natural frequency, and frequency response were calculated. The feasibility and superiority of the sensing design were verified through experiments. Compared with the standard product 3M electronic stethoscope, the results showed that the heart sound signal collected by the MEMS bionic sensor was of higher quality, which provides ideas for designing high-performance electronic stethoscopes in the future, and the development of artificial medical intelligence.

## Figures and Tables

**Table 1:** The Description of Microstructure Parameters.

Parameter	Description	Actual Value (mm)
*a*	Length of column	0.6
*b*	Width of cantilever	0.12
*c*	Length of mass	0.6
*d*	Thickness of cantilever	0.04
*h*	Hight of column	0.8
*l*	Length of cantilever	1
*r*	Radius of otolith	2.4

**Table 2:** Comparison of various material parameters.

Material	Density (kg/m^3^)	Speed of Sound (m/s)	Characteristic Impedance (Pa/m^2^∙s)
Air	1.205	340	420
Water	1000	1500	1.48 × 10^6^
Body	1020	1540	1.4 × 10^6^~1.7 × 10^6^
Castor oil	955–970	1477	1.45 × 10^6^

**Table 3:** Data of MEMS sensor and 3M electronic stethoscope.

Type	MEMS Sensor		3M Electronic Stethoscope	
	V_s_(mv)	V_n_(mv)	V_s_(mv)	V_n_(mv)
Peak-to-Peak	418.3	2.325	291.4	2.119
RMS	28.63	0.334	22.68	0.298
SNR (dB)	38.6		37.6	

**Table 4:** Comparison of SNR of sensors with different structure.

Types	SNR
Cilium and Sphere	15.1
Cilium	18.64
Lollipop	25.72
Bat	29.08
Fish ear	38.6

## Data Availability

Data is contained within the article.
